# Comparing Auto-Machine Learning and Expert-Designed Models in Diagnosing Vitreomacular Interface Disorders

**DOI:** 10.3390/jcm14082774

**Published:** 2025-04-17

**Authors:** Ceren Durmaz Engin, Mahmut Ozan Gokkan, Seher Koksaldi, Mustafa Kayabasi, Ufuk Besenk, Mustafa Alper Selver, Andrzej Grzybowski

**Affiliations:** 1 Department of Ophthalmology, Izmir Democracy University Buca Seyfi Demirsoy Education and Research Hospital, Izmir 35390, Turkey; 2 Izmir Health Technologies Development and Accelerator (BioIzmir), Dokuz Eylul University, Izmir 35330, Turkey; ozangokkan@gmail.com (M.O.G.); alper.selver@deu.edu.tr (M.A.S.); 3 Department of Ophthalmology, Mus State Hospital, Mus 49200, Turkey; skayabasi@agri.edu.tr (S.K.); mkayabasi94@gmail.com (M.K.); 4 Department of Electrical and Electronics Engineering, Dokuz Eylul University, Izmir 35390, Turkey; ufuk.besenk@ogr.deu.edu.tr; 5 Department of Ophthalmology, University of Warmia and Mazury, ul. Warszawska 30, 10-082 Olsztyn, Poland; 6 Institute for Research in Ophthalmology, Fundacja Wspierania Rozwoju Okulistyki “Okulistyka 21”, ul. Mickiewicza 24/3B, 60-836 Poznań, Poland

**Keywords:** AutoML, deep learning, EfficientNet B0, optical coherence tomography, ResNet-50, vitreomacular interface disorders

## Abstract

**Background:** The vitreomacular interface (VMI) encompasses a group of retinal disorders that significantly impact vision, requiring accurate classification for effective management. This study aims to compare the effectiveness of an expert-designed custom deep learning (DL) model and a code free Auto Machine Learning (ML) model in classifying optical coherence tomography (OCT) images of VMI disorders. **Materials and Methods:** A balanced dataset of OCT images across five classes—normal, epiretinal membrane (ERM), idiopathic full-thickness macular hole (FTMH), lamellar macular hole (LMH), and vitreomacular traction (VMT)—was used. The expert-designed model combined ResNet-50 and EfficientNet-B0 architectures with Monte Carlo cross-validation. The AutoML model was created on Google Vertex AI, which handled data processing, model selection, and hyperparameter tuning automatically. Performance was evaluated using average precision, precision, and recall metrics. **Results:** The expert-designed model achieved an overall balanced accuracy of 95.97% and a Matthews Correlation Coefficient (MCC) of 94.65%. Both models attained 100% precision and recall for normal cases. For FTMH, the expert model reached perfect precision and recall, while the AutoML model scored 97.8% average precision, and 97.4% recall. In VMT detection, the AutoML model showed 99.5% average precision with a slightly lower recall of 94.7% compared to the expert model’s 95%. For ERM, the expert model achieved 95% recall, while the AutoML model had higher precision at 93.9% but a lower recall of 79.5%. In LMH classification, the expert model exhibited 95% precision, compared to 72.3% for the AutoML model, with similar recall for both (88% and 87.2%, respectively). **Conclusions:** While the AutoML model demonstrated strong performance, the expert-designed model achieved superior accuracy across certain classes. AutoML platforms, although accessible to healthcare professionals, may require further advancements to match the performance of expert-designed models in clinical applications.

## 1. Introduction

Vitreomacular interface (VMI) disorders represent a spectrum of retinal pathologies characterized by alterations at the interface between the vitreous and the macula, an area crucial for central vision and detailed visual processing. Among these disorders, epiretinal membrane (ERM), idiopathic full-thickness macular hole (FTMH), lamellar macular hole (LMH), and vitreomacular traction (VMT) are commonly encountered in clinical practice and can lead to progressive visual deterioration if left untreated. Accurate diagnosis and differentiation of VMI disorders are clinically significant, as they directly inform therapeutic decisions, which may range from conservative monitoring to surgical intervention. Optical coherence tomography (OCT) serves as the preferred imaging modality for evaluating VMI pathologies due to its high-resolution, cross-sectional visualization of retinal microstructures, enabling precise assessment of the macular layers and pathological changes within the vitreoretinal interface.

Deep learning (DL) has shown exceptional potential in automating the analysis of OCT images, achieving high diagnostic accuracy and reliability in detecting and classifying retinal conditions, including VMI disorders. Valentim et al. [1] achieved 88.5% accuracy in detecting FTMH and identifying specific stages through a DL-based algorithm. Similarly, Xiao et al. [2] developed a DL model that accurately classified macular hole etiology, distinguishing idiopathic from secondary macular holes, with an external test set yielding an area under the receiver operating characteristic curve (AUC) of 0.965. Furthermore, this model effectively predicted postoperative anatomical outcomes, achieving high predictive accuracy for macular hole closure status following surgery, thus underscoring the model’s potential in clinical decision-making for FTMH management. However, the development of such models traditionally requires expertise in coding, which limits their accessibility to a subset of artificial intelligent (AI) specialists. To address this gap, code-free Auto Machine Learning (ML) platforms have been developed, enabling clinicians without programming skills to create ML models. Several studies have evaluated the performance of different AutoML platforms in ophthalmic diseases including diabetic retinopathy grading, differentiating cataract surgery phases and retinal multi-disease classification [3,4,5]. Recently, in a study by Kırık et al. [6], a code-free ML model developed using Teachable Machine (version 2.0) achieved 100% sensitivity and 100% specificity in the classification of VMI disorders, including ERM, VMT, and FTMH, with an AUC of 0.99 for ERM detection and 0.97 for FTMH detection. Although promising, AutoML models still face challenges in achieving consistent performance across diverse datasets and ensuring clinical robustness, particularly in complex ophthalmic cases [7].

Therefore, this study aims to evaluate and compare the performance of two distinct DL models—a code free AutoML and an expert-designed custom DL model—in identifying VMI disorders compared to normal OCT images, and classifying them. To our knowledge, this study is the first to explore and compare the diagnostic capabilities of those two models in VMI disorder classification, offering valuable insights into the clinical accessibility of AutoML as a diagnostic tool for ophthalmologists.

## 2. Materials and Methods

### 2.1. Building the Dataset

The dataset was retrospectively compiled from OCT images of patients aged 18 years and older who presented to our tertiary ophthalmology clinic between September 2020 and September 2024. Exclusion criteria included the presence of prominent retinal pathologies other than VMI disorders, secondary causes of FTMH, OCT images with artifacts or noise reducing signal strength below 30, and any history of vitreoretinal surgery. For analysis, 200 images were selected for each VMI disorder—ERM, FTMH, and LMH—while 100 images were included for VMT. Additionally, a control group comprised 200 normal OCT images from patients without retinal pathology. Diagnostic classification for each group was determined by consensus among three experienced ophthalmologists (C.D.E., M.K., and S.K.) according to an OCT-based anatomic classification system developed by the International Vitreomacular Traction Study Group [8]. All images were acquired using the Topcon Triton OCT device (Topcon Medical Systems, Tokyo, Japan) with radial scanning protocols. To standardize the dataset, only OCT images of right eyes were included and, for optimal visualization of the VMI, only horizontal line scans centered on the macula were utilized. To maintain uniformity in image quality and resolution across both models, all images were cropped and set to 300 dpi for use.

This cross-sectional study was approved by the Institutional Review Board of Buca Seyfi Demirsoy Education and Research Hospital (Approval date: 22 November 2024 and number: 2024/357), and informed consent was waived as the images used did not contain any identifiable patient information. The study was conducted in accordance with the principles outlined in the Declaration of Helsinki.

### 2.2. Designing Expert Model

#### 2.2.1. Dataset and Image Preprocessing

For training and validation, 160 images were archived per class for ERM, FTMH, LMH, and normal categories, with 80 images for VMT. The test set contained 40 images each for ERM, FTMH, LMH, and normal categories, and 20 images for VMT. Data augmentation techniques, including horizontal flipping, limited shift-scale-rotation (rotation limit: 2°), contrast-limited adaptive histogram equalization (clip limit: 2), random brightness contrast adjustments, and Gaussian noise addition, were applied. A 2-fold cross-validation technique was employed, leveraging the balanced dataset.

#### 2.2.2. Model Architecture and Feature Extraction

A novel approach combining the ResNet-50 and EfficientNet-B0 models [9,10,11,12] was implemented to benchmark the performance comparison between the expert-designed model and AutoML models. To achieve this, ImageNet-pretrained weights for both ResNet-50 and EfficientNet-B0 were transferred to our deep neural network model, with the final fully connected layer modified to classify five classes. These pretrained weights were initialized at the top of our model, and training was conducted without freezing any layers, allowing all learnable parameters to be updated during backpropagation. ResNet-50, a convolutional neural network (CNN) architecture with 50 convolutional layers and residual blocks, and EfficientNet-B0, with mobile-inverted bottleneck (MBConv) blocks, were selected for feature extraction based on model complexity, inference time, and performance metrics. ResNet-50 produced 2048 features in its final layer, while EfficientNet-B0 generated 1280 features. These combined features were input into a fully connected layer, where classification was achieved using a ReLU activation function followed by linear transformation, resulting in a five-class output.

#### 2.2.3. Cross-Validation and Monte Carlo Sampling

**Figure 1:**
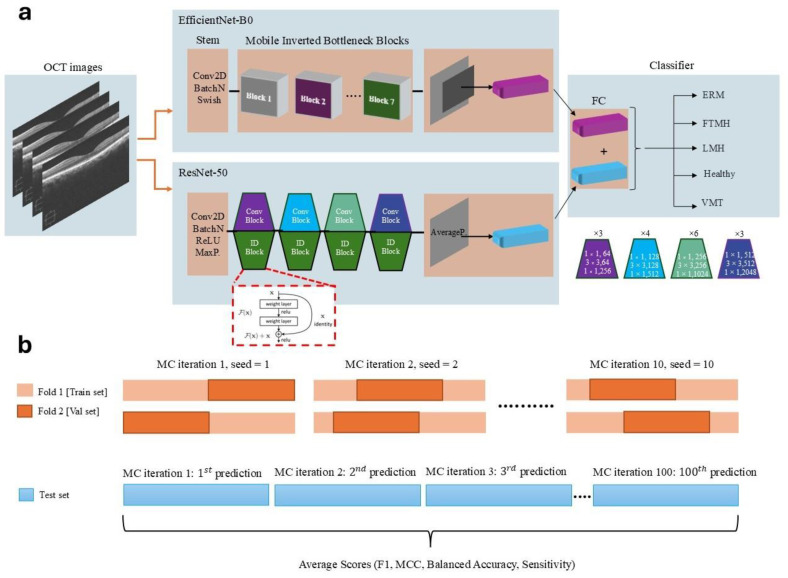
(**a**) Overview of the dual-path convolutional neural network model architecture, combining EfficientNet-B0 and ResNet-50 for feature extraction and classification of OCT images into five categories: ERM, FTMH, LMH, Normal, and VMT. (**b**) Illustration of the Monte Carlo cross-validation process, demonstrating dataset splits and the iterative process used for performance metric averaging across multiple test predictions.

In deep CNNs, random sampling or random number generation can occur during both the training and testing phases, depending on the model design. During training, a probabilistic approach is often employed in the dropout layer and data augmentation processes. In the dropout layer, a specified probability value (e.g., *p* = 0.5) determines the fraction of neurons to be randomly dropped during each forward pass. For instance, with *p* = 0.5, 50% of the neurons are randomly deactivated in each forward pass. Similarly, when applying data augmentation techniques to images, these techniques are applied probabilistically, based on the specified value of *p*. In the testing phase, predictions are made using the trained CNN model, and performance metrics are typically calculated by averaging predictions over multiple runs or ensembles, providing a more robust evaluation of the model’s performance. Considering the balanced class distribution, KFold cross-validation was employed instead of stratified KFold, commonly used for imbalanced datasets. To further enhance the model’s robustness and prevent overfitting, a Monte Carlo (MC) cross-validation approach with 10 iterations was used [13]. Each iteration included unique random seed values, ensuring varied indexing for the training and validation sets. This MC cross-validation technique allowed for ensemble averaging across multiple samples, yielding more reliable statistical estimations. Since both k-fold cross-validation and the Monte Carlo technique were applied simultaneously, the training phase was completed using 2-fold cross-validation and 10 Monte Carlo iterations, which were determined experimentally as the optimal values. During external testing, model predictions were stored in arrays across 100 MC iterations, and the average values of performance metrics were calculated to produce the final test scores. The detailed architecture of the expert-designed model is presented in Figure 1.

#### 2.2.4. Training Procedure

The model was trained on OCT images as input to both ResNet-50 and EfficientNet-B0, with the combined features subsequently processed in the fully connected layer for classification. Multi-class cross-entropy loss was utilized, with the AdamW optimizer configured with a learning rate (LR) and weight decay of 1 × 10^−4^. Training was performed on an ASUS ProArt workstation (ASUS, Taipei, Taiwan) equipped with an NVIDIA GeForce RTX 4090 GPU (NVIDIA Corporation, Santa Clara, CA, USA) and an Intel Core i9-13900 CPU (Intel Corporation, Santa Clara, CA, USA; 32 CPUs). Training was limited to a maximum of 100 epochs, with early stopping applied if validation loss did not improve for 10 consecutive epochs, significantly reducing the total training time. In our study, tunable hyperparameters were optimized based on both experimental results and the hardware capacity of our workstation. For instance, the gradient accumulation (GA) technique was employed to update the model’s weights after accumulating gradients over multiple mini-batches, rather than after each individual mini-batch during backpropagation. Specifically, the parameter accumulation_steps = batch_size/len(train_loader) was used to ensure that gradient accumulation was performed in accordance with the batch size and dataset size. This approach optimized memory usage efficiency, enabling training with a batch size of 32 without exceeding memory limits.

The AdamW optimizer was used with LR of 1 × 10^−4^, a weight decay of 1 × 10^−4^, and default beta momentum values of 0.9 and 0.999. A small LR was chosen to facilitate convergence to the global minimum of the loss function. For LR scheduling, the ReduceLROnPlateau scheduler in PyTorch (ver. 2.1.0+cu118) was utilized to dynamically adjust the LR based on validation loss. The objective was to reduce the LR when validation loss plateaued, helping the model converge to a better minimum while mitigating the risk of overfitting or becoming stuck in a suboptimal solution. In this study, if the validation loss did not decrease for three consecutive epochs, the LR was reduced by a factor of 0.1, allowing for finer adjustments during training.

### 2.3. Designing AutoML Model

#### 2.3.1. Dataset Preparation

The same OCT image dataset used in the expert model, including balanced samples across five classes (ERM, FTMH, LMH, VMT and normal images), was uploaded to Google Cloud. Images were prepared in both training and test sets, with the Vertex AI AutoML platform handling an 80-20 split (training and validation, and test) as part of its default configuration. Both the custom DL and AutoML models were trained and tested using the same set of images for their respective training and testing datasets.

#### 2.3.2. Model Development

Using Google Vertex AI AutoML Model training was conducted on Google Vertex AI AutoML Vision, a platform optimized for image classification tasks with a fully automated pipeline by an ophthalmologist. In Vertex, the multi-level image classification task was selected, and default settings were applied in subsequent steps, allowing the platform to handle feature extraction, model selection, and hyperparameter tuning to determine the optimal architecture and training configurations. AutoML’s transfer learning framework leveraged pre-trained convolutional neural networks, while Bayesian optimization refined key hyperparameters, including LR and batch size.

#### 2.3.3. Training and Optimization

The Vertex AI training environment leveraged Google’s tensor processing units to accelerate the computation process. The platform applied default data augmentation techniques, including rotations, brightness adjustments, and contrast modifications, enhancing model robustness. Additionally, early stopping criteria were enforced to prevent overfitting, halting training when validation loss did not improve over several epochs.

### 2.4. Evaluation Metrics

Both models underwent detailed performance evaluation to ensure reliable and generalizable results in OCT image classification. Train loss, validation loss, train accuracy, and validation accuracy were recorded across Monte Carlo (MC) iterations, providing robust performance tracking in the expert model. Key metrics for both models—including average precision, precision, and recall—were calculated for each class, along with confusion matrices to assess classification accuracy across all OCT categories. Precision was defined as the ratio of correctly predicted positive observations (true positives) to the total predicted positives (true positives + false positives). Recall (also known as sensitivity) represented the ratio of correctly predicted positive observations (true positives) to all observations in the actual positive class (true positives + false negatives). Average precision summarized the precision–recall curve by computing the weighted mean of precision values achieved at different recall levels, providing a comprehensive measure of model performance across varying thresholds. To interpret the expert model’s predictions and visualize the regions contributing to the classification of VMI disorders, Gradient-weighted Class Activation Mapping (Grad-CAM) was applied.

## 3. Results

### 3.1. Expert-Designed Model

The expert-designed model exhibited strong performance across all categories, achieving an overall Matthews Correlation Coefficient (MCC) of 94.65%. The balanced accuracy score reached 95.97%, with an overall recall score of 95.82%, indicating the model’s high reliability in accurately distinguishing between different VMI disorders.

When examining the class-specific metrics, the model attained 100% precision and recall for the Normal and FTMH classes, demonstrating flawless classification in these categories. Similarly, the VMT category achieved perfect precision and a high recall of 95%, reflecting the model’s strong ability to detect VMT cases with minimal false negatives. For the ERM class, the model achieved a precision of 86% and a recall of 95%, indicating some misclassification but still performing well overall. The LMH class showed precision at 95% and recall at 88%, demonstrating effective detection with a slight drop in recall compared to other classes.

**Figure 2:**
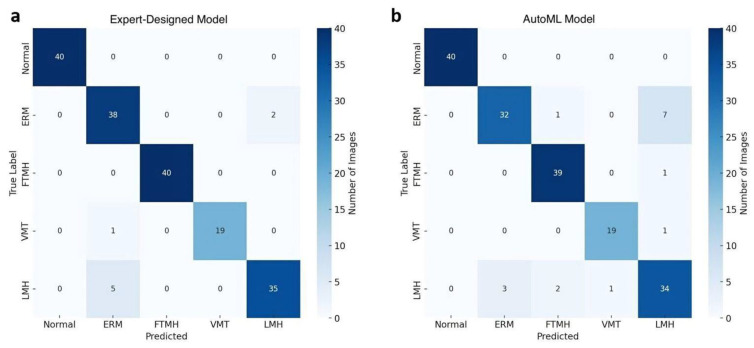
Confusion matrices comparing the performance of the expert-designed model (**a**) and the AutoML model (**b**) in classifying Normal and vitreomacular interface disorders on OCT images.

The confusion matrix, depicted in Figure 2a, provides further insights into model predictions across each category. Notably, a few ERM cases were misclassified as LMH, reflecting potential overlap in visual features between these categories. However, the model consistently classified Normal, FTMH, and VMT categories with high accuracy, with no misclassifications in these groups. The performance metrics are summarized in Table 1.

**Figure 3:**
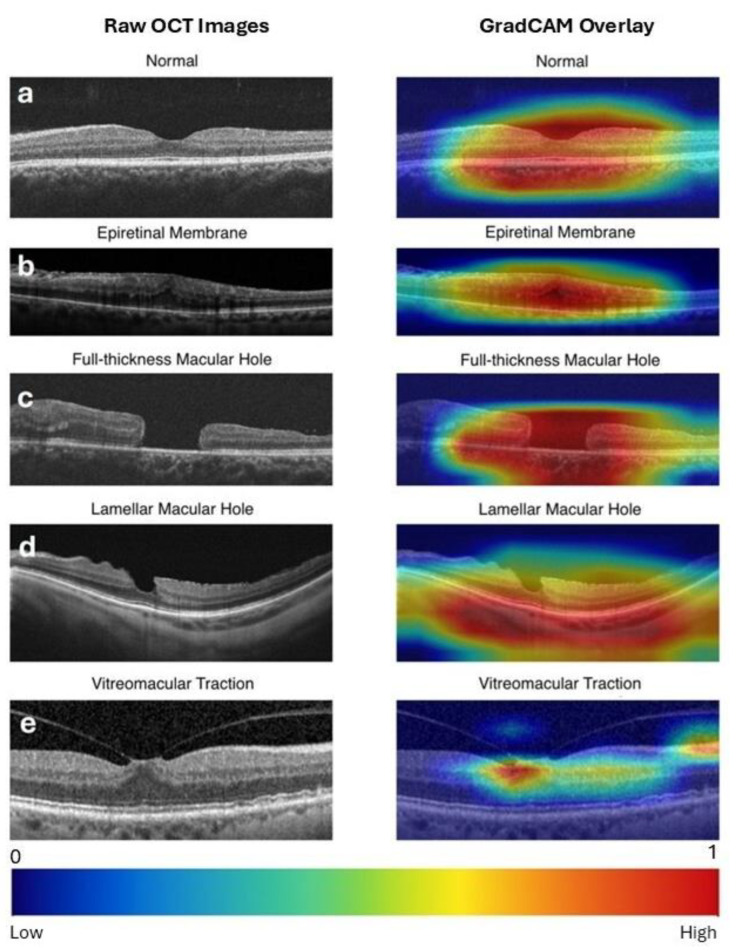
Raw optical coherence tomography (OCT) images (left column) and Gradient-weighted Class Activation Mapping (Grad-CAM) visualizations of the expert-designed model predictions (right column) for vitreomacular interface disorders. Grad-CAM highlights the regions of the image that contribute most to the model’s prediction. Warmer colors (e.g., red) indicate higher importance, while cooler colors (e.g., blue) reflect minimal influence. A scale bar is provided at the bottom for reference. (**a**) Normal: Activation highlights the central retinal area, confirming the absence of pathology. (**b**) ERM (Epiretinal Membrane): Strong activation over a broad retinal area, rather than the foveal center, corresponds to the presence of epiretinal tissue extending across the retina. (**c**) FTMH (Full-Thickness Macular Hole): Intense focus on the macular defect accurately identifies the location of the hole. (**d**) LMH (Lamellar Macular Hole): Activation captures the partial macular defect. (**e**) VMT (Vitreomacular Traction): Heatmaps emphasize the vitreomacular attachment point, reflecting the model’s identification of traction-related features.

Grad-CAM generated a heatmap overlay on the original image by highlighting the most influential regions responsible for the model’s decisions, such as the macular hole region for FTMH and the vitreomacular attachment point for VMT. Representative Grad-CAM visualizations for each class are presented in Figure 3.

### 3.2. AutoML Model

The model demonstrated robust performance across all labels, achieving an overall average precision of 95.9%. Precision and recall were measured at 89.9% and 91.4%, respectively.

In terms of label-specific performance, the model achieved 100% precision and recall for the Normal category. Both FTMH and VMT categories also demonstrated high accuracy, with average precision of 97.8% and 96.0%, respectively. However, the ERM category displayed relatively lower recall at 79.5%, indicating some degree of misclassification within this category. The LMH category also showed a slightly reduced performance, with a precision of 72.3% and a recall of 87.2%. The precision and recall values for each class are summarized in Table 1.

A detailed examination of model predictions is presented in the confusion matrix in Figure 2b. This matrix reveals that ERM cases were occasionally misclassified as LMH (18%) and FTMH (3%), whereas misclassifications were minimal for the Normal, FTMH, and VMT categories.

## 4. Discussion

This study compares the performance of an AutoML model developed by an ophthalmologist with that of a custom DL expert model created by an engineer for the recognition and classification of VMI interface disorders from OCT images and, to our knowledge, represents the first study in the literature to conduct such a comparison. Although both models demonstrated improved accuracy, particularly in identifying cases with more prominent image features, such as normal eyes, FTMH, and VMT, the expert model outperformed the AutoML model in both binary and multiclass image recognition tasks.

In their study comparing bespoke and AutoML models for classifying OCT images of normal retina, FTMH, ERM, wet age-related macular degeneration, and diabetic macular edema, Touma et al. [14] observed that the AutoML model achieved classification accuracies of 96.2% for normal cases, 91.7% for MH cases, and 90.5% for ERM cases. Upon generating saliency maps, they determined that this performance was primarily attributed to the model’s focus on the presence of a continuous external limiting membrane and retinal pigment epithelium. Although our study involved a different dataset and disease groups, the “Normal OCT” category similarly emerged as the group with the highest classification accuracy in both models in our study, which can be attributed to the distinct visual features of healthy OCT images, which lack pathological variations, making them easier for both the expert-designed and AutoML models to identify without misclassification.

In the FTMH class, the expert-designed model achieved 100% precision and recall, while the AutoML model performed slightly lower, with a precision of 92.7% and recall of 97.4%. The superior performance of the expert-designed model may be attributed to its dual-pathway architecture, which leverages diverse feature extraction through EfficientNet and ResNet layers, compared to the AutoML model’s more general approach. In a recent study by Valentim et al. [1], a built-in algorithm called B-Scans of Interest in an OCT device achieved an accuracy of 91.4% in identifying OCT features of FTMHs compared to posterior vitreous detachment cases used as controls. This model was trained with 76,800 SD-OCT images containing multiple retinal disorders. Although our models were trained with far fewer images, the incorporation of image augmentation in both of our models, the transfer learning component of the AutoML model, and the ensemble architecture of the expert-designed model likely contributed to their high performance. As demonstrated in previous literature, excessively similar data points can lead to overfitting, where the model memorizes redundant features rather than learning generalized patterns [15]. Previous literature also emphasizes that data diversity is more critical than sheer data volume for training robust models [16]. By training on a diverse yet sufficient number of images, it is possible to achieve better generalizability and performance [17,18]. This point is further supported by a study conducted by Xiao et al. [2], where a DL model was trained and tested using 1082 OCT images from 330 eyes to classify MH etiology. Despite the smaller dataset size, the algorithm achieved an AUC of 0.965 and accuracy of 0.950. in successfully differentiating idiopathic (primary) MHs from secondary MHs, a more challenging task compared to distinguishing macular holes from other VMI abnormalities.

In our study, the expert-designed model and the AutoML model both demonstrated high classification performance for VMT, with the expert model achieving 100% precision and 95% recall, while the AutoML model attained 100% precision but a slightly lower recall of 94.7%. These findings are consistent with previous research by Kirik et al. [6], which showed that a code-free AI model using Teachable Machine achieved 98.08% specificity and an AUC of 0.99 for VMT detection. Additionally, Usmani et al. [19] highlighted the potential of DL models in predicting VMT outcomes, with a positive predictive value of 0.79 for stable cases. While our results confirm that both expert-designed and AutoML models can reliably identify VMT, further refinement of AutoML approaches may improve their accuracy in clinical practice.

Both the expert-designed model and the AutoML model demonstrated the lowest classification performance for ERM and LMH. To date, several studies have evaluated the use of DL algorithms in ERM in terms of recognition, classification, prediction of visual impairment, and the prediction of postoperative visual and anatomical outcomes following vitrectomy surgery. These studies have reported ERM detection accuracies ranging from 70% to 99.7% [9,10,11,12]. In present study, the expert-designed model achieved a recall of 95% but lower precision of 86%, whereas the AutoML model had a higher precision of 93.9% but lower recall of 79.5% in the ERM category. The expert model’s higher recall may suggest it was better at detecting ERM cases but at the cost of some false positives. This could be due to its tailored preprocessing techniques, which enhanced sensitivity to ERM-specific texture changes compared to the AutoML model. The AutoML model’s higher precision might reflect its more generalized feature extraction, which was effective in ERM detection but may have missed subtler variations, leading to lower recall. While, to our knowledge, no study has evaluated the detection of LMH within a cohort of VMI disorders, a recent study utilized a pretrained DL model of Inception-ResNet-v2 to identify imaging biomarkers associated with the anatomic and functional progression of LMHs, demonstrating an accuracy of 92.5% in predicting functionally progressing LMHs [20]. In the present study, the expert model significantly outperformed the AutoML model in precision (95% vs. 72.3%), while both had similar recall rates (88% and 87.2%, respectively). The expert model’s advantage in precision could be due to its use of Monte Carlo cross-validation, which helped reduce overfitting and provided more robust class distinctions. LMH features can often be confused with other disorders, most commonly with ERM and FTMH, and the expert model’s feature aggregation from dual CNNs is likely to have contributed to clearer differentiation.

AutoML models rely on generalizable feature extraction and transfer learning from pre-trained CNN. In our study, AutoML demonstrated higher performance in FTMH and VMT, likely due to the clear morphological characteristics of these conditions. FTMH presents a distinct full-thickness defect in the foveal region, and VMT is characterized by a well-defined vitreomacular attachment, making them visually prominent even for non-specialized algorithms. In contrast, ERM and LMH exhibit more subtle and overlapping features, where fine structural differences play a crucial role in differentiation. Expert-designed models, with tailored preprocessing and feature engineering, may capture these nuanced details better than AutoML.

The combination of ResNet-50 and EfficientNet-B0 architectures can enhance model performance by leveraging their complementary strengths. ResNet-50 excels in extracting complex, high-level features through its residual connections, which maintain learning efficiency in deep networks. EfficientNet-B0, on the other hand, achieves high accuracy with optimized computational efficiency, making it particularly effective for capturing fine-grained details. Together, these architectures enable the model to process a broader range of features, combining hierarchical and detailed spatial information. The literature supports the use of multi-model approaches for improving performance in medical images. ResNet-50 is widely recognized in medical imaging for its ability to extract intricate patterns, while EfficientNet-B0 demonstrates strong performance in tasks requiring sensitivity to subtle details with lower computational demands. By combining these architectures, the model can reduce overfitting and achieve better generalizability, even in scenarios with limited datasets [21,22]. For instance, a recent study demonstrated that an ensemble of deep CNNs trained on 43,055 retinal fundus images from 12 public datasets outperformed board-certified ophthalmologists in classifying diabetic retinopathy, glaucoma, age-related macular degeneration, and normal eyes, achieving a mean accuracy of 79.2% compared to 72.7% (*p* = 0.03), with statistically significant higher F1-scores for diabetic retinopathy (76.8% vs. 57.5%; *p* = 0.01) and a greater agreement between accuracy and confidence (81.6% vs. 70.3%; *p* < 0.001) [23]. Another study that used similar CNN architectures in our study, highlighted the effectiveness of an ensemble model combining ResNet50 and EfficientNet-B7 for brain tumor classification, achieving superior validation accuracy of 99.68% compared to ResNet50 (97.65%) and EfficientNet-B7 (98.20%) when trained on high-resolution MRI images across four classes (no tumor, glioma, meningioma, and pituitary tumor), with post-training analysis further confirming its robustness at 99.53% accuracy, significantly outperforming the individual models [24].

In this study, diagnostic classification was determined by consensus among three experienced ophthalmologists, with inter-observer variability occurring in only 12 out of 1100 cases (1.09%). These discrepancies were resolved by structured discussions referencing the international OCT-based classification system, minimizing potential mislabeling. While such minimal variability likely had negligible effects on our models’ precision and recall, it is crucial to recognize that, if inter-observer variability were significantly higher, this could lead to incorrect labeling of training and test datasets, adversely impacting model performance. Therefore, ensuring high reliability and accurate labeling remains essential for developing robust and clinically applicable AI models.

The limitations of this study include the use of a relatively small dataset, which may constrain the generalizability and accuracy of the models. The inclusion of exclusively idiopathic FTMH cases, while excluding images with concurrent pathologies frequently encountered in clinical practice, such as pathological myopia, further restricts the algorithm’s generalizability and applicability. Another limitation is the restricted customizability of the AutoML model, as Google Vertex AI does not provide access to the underlying architecture or code. This constraint prevented any component-level modification or integration of expert-designed model features into the AutoML pipeline. Additionally, the reliance on images acquired from a single OCT device at a single medical institution, coupled with the cross-sectional design of the study, limits the broader applicability of the findings. Future research involving larger, more diverse datasets and images from multiple devices and institutions is needed to validate and extend these results.

## 5. Conclusions

This study is significant as it is the first to directly compare an expert-designed model with an AutoML platform for OCT-based classification of VMI disorders. Overall, the expert-designed model outperformed the AutoML model, likely due to its sophisticated architecture, which integrates two CNNs for enhanced feature extraction, and its comprehensive preprocessing techniques, including augmentation and Monte Carlo cross-validation. These strategies are likely to have enabled the model to capture finer details in OCT images, thereby improving its ability to distinguish between VMI disorders. However, both models demonstrated strong classification performance across VMI disorders, with the expert model excelling in more complex and overlapping cases such as ERM and LMH, while the AutoML model performed competitively in conditions with distinct morphological features, like FTMH and VMT. This highlights the potential of both approaches in automated VMI classification, depending on the complexity of the pathology. The AutoML model, while leveraging default data augmentation and transfer learning from pre-trained models, may not be specifically optimized for VMI disorder classification. The acceptable performance of the AutoML models in this study highlights their potential for clinical applications by individuals with no coding experience, while also emphasizing the current advantages of expert-crafted architectures in addressing specialized imaging tasks.

## Figures and Tables

**Table 1:** Class-specific performance metrics of Expert and Auto Machine Learning models in classification of vitreomacular interface disorders.

	Average Precision		Precision (%)		Recall (%)	
	Expert	AutoML	Expert	AutoML	Expert	AutoML
Normal	1.000	100.0	100.0	100.0	100.0	100.0
FTMH	100.0	97.8	100.0	92.7	100.0	97.4
LMH	90.3	86.0	95.0	72.3	88.0	87.2
ERM	95.3	83.0	86.0	93.9	95.0	79.5
VMT	99.5	96.0	100	100	95.0	94.7

## Data Availability

The dataset is available upon request from the corresponding author.

## Abbreviations

The following abbreviations are used in this manuscript:

AI	Artificial Intelligence
AUC	Area Under the Receiver Operating Characteristic Curve
CNN	Convolutional Neural Network
DL	Deep Learning
ERM	Epiretinal Membrane
FTMH	Full-Thickness Macular Hole
Grad-CAM	Gradient-weighted Class Activation Mapping
LMH	Lamellar Macular Hole
MC	Monte Carlo
MCC	Matthews Correlation Coefficient
ML	Machine Learning
OCT	Optical Coherence Tomography
VMI	Vitreomacular Interface
VMT	Vitreomacular Traction
